# A Rare Case of Single-Rooted Mandibular Second Molar with Single Canal

**DOI:** 10.1155/2020/8096539

**Published:** 2020-06-15

**Authors:** Abdulmohsen Alfadley, Ahmad Alquraishi, Yaser Almazrou, Fahd Aljarbou

**Affiliations:** ^1^Department of Restorative and Prosthetic Dental Sciences, College of Dentistry, King Saud bin Abdulaziz University for Health Sciences, Riyadh, Saudi Arabia; ^2^King Abdullah International Medical Research Center, Ministry of National Guard Health Affairs, Riyadh, Saudi Arabia; ^3^Internship Unit, College of Dentistry, King Saud bin Abdulaziz University for Health Sciences, Ministry of National Guard Health Affairs, Riyadh, Saudi Arabia; ^4^Presidency of State Security, Department of Medical Services, Riyadh, Saudi Arabia; ^5^Department of Restorative Dental Sciences, Division of Endodontics, College of Dentistry, King Saud University, Riyadh, Saudi Arabia

## Abstract

The root canal anatomy of mandibular second molar teeth is known to be highly variable. Whilst the most frequently seen configuration is two mesial canals and one distal canal, other variations such as four canals, two canals, and C-shaped canal system do also exist. This case report describes the diagnosis and management of unusual root canal configuration of a mandibular second molar, with one canal in a single conical root, using the contemporary advancements in endodontics. Following clinical and radiographic examinations of the case, a diagnosis of symptomatic irreversible pulpitis with symptomatic apical periodontitis of tooth #47 was established, and root canal treatment followed by composite buildup and crown were planned. Clinicians should be aware of the different anatomic variants each tooth may exhibit. Furthermore, clinicians need to possess the proper knowledge and skills that allow them to utilize the diagnostic and therapeutic tools available at their disposal in order to optimize the quality of care provided to their patients.

## 1. Introduction

The principal objective of endodontic treatment is to prevent and, when needed, to treat endodontic disease, apical periodontitis [1]. In order to accomplish this objective, high-quality chemomechanical cleaning and shaping of the intricate root canal system are required in order to eradicate intracanal microorganisms [2, 3]. This task requires elaborate knowledge of pulp space anatomy, including expected and less usual canal morphology. Variations such as additional canals, bi/trifurcations, lateral and furcal canals, apical deltas, and canal ramifications are frequently encountered during root canal treatment, and their incidence and clinical significance have been reported before [4, 5]. Due to the complex and highly variable pulp space anatomy, it is not uncommon to overlook part of this anatomy during the debridement phase [6] resulting in the emergence or persistence of endodontic disease [7].

The root canal morphology of mandibular second molars has been elegantly evaluated in landmark studies by Vertucci and Weine et al. using clearing and radiographic techniques, respectively [8, 9]. Similar to the mandibular first molar, this tooth most commonly has two roots; mesial and distal, with two mesial canals (mesiobuccal and mesiolingual) and one distal canal [8–10]. Nevertheless, other configurations have been reported in mandibular second molar teeth such as four canals, two canals, C-shaped canal, taurodontism, and one canal in a single root [8–12]. Previously published articles agree on the rare occurrence of one canal in a single-rooted (Vertucci Type I) mandibular second molars. For instance, Weine et al. reported that only 1.3% of mandibular second molars had a single canal configuration all the way from an orifice to an apex [9]. In line with that, Demirbuga et al. used advanced imaging technique and found the prevalence of such anatomic variant to be close to 2% [13]. Such high variability in the root canal anatomy of this tooth emphasizes the importance of properly integrating theoretical knowledge of dental morphology with the information obtained from pretreatment radiographs. Furthermore, additional exploration of internal anatomy should be made during treatment in order to avoid the possibility of untreated canal system. Several of the recent advances in endodontics have contributed to safe, predictable, and efficient root canal treatment. Such developments include digital radiography, cone beam computed tomography (CBCT), microcomputed tomography (micro-CT), dental operating microscope (DOM), thermomechanically treated nickel-titanium files, and improved obturation devices [14–17]. The aim of this case report was to describe the diagnosis and management of rare root canal configuration of a mandibular second molar, showing one canal in a single conical root, using the contemporary advancements in endodontics.

## 2. Case Report

A 25-year-old Saudi Arabian man presented to the dental clinic with a chief complaint of “severe pain to cold water” in the lower right side of the jaw. The history revealed that pain started about three weeks ago and has increased in intensity over the past five days with occasional spontaneous symptoms. The patient had several dental treatments done in the past such as periodontal scaling, restorations, root canal treatment, and extractions, without any complications. The patient denied having any relevant medical condition such as chronic illness, use of medication, allergy, or previous hospitalization.

**Figure 1:**
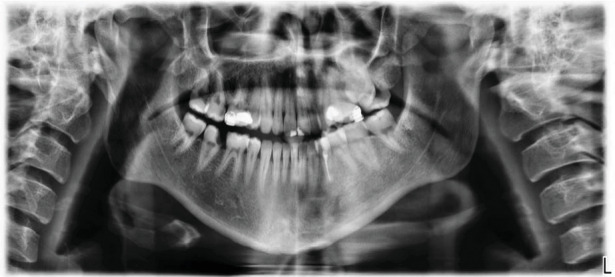
Panoramic radiograph of presenting patient confirms the lack of symmetry between single-rooted tooth #47 and contralateral tooth #37 which presents with two distinct roots.

**Figure 2:**
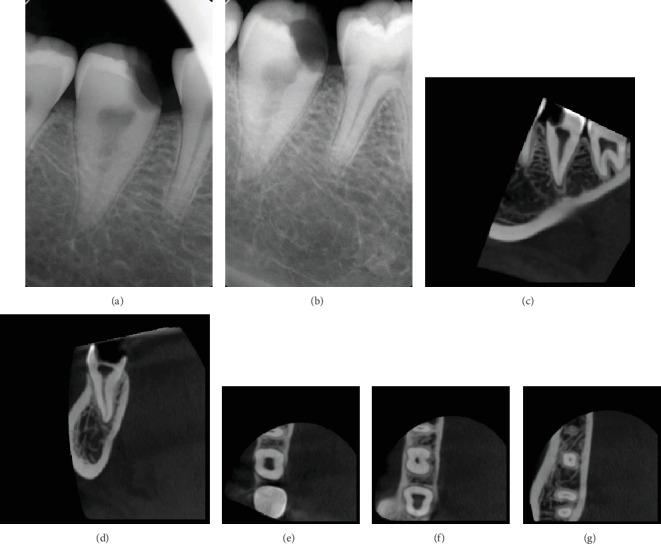
Preoperative radiographic assessment. (a) Straight-on and (b) angulated initial radiographs showing tooth #47 with deep occluso-mesial caries along with large pulp space situated within a single conical root. (c, d) Sagittal and coronal CBCT slices suggest the presence of one root canal configuration. (e–g) Further evaluation of the axial cuts in the coronal, middle, and apical thirds confirms the presence of Vertucci Type I canal system.

Upon extraoral examination, no significant findings were noted. Intraoral examination showed tooth #47 with deep occluso-mesial caries. On application of the cold test to the tooth, the patient responded with severe prolonged pain compared to adjacent teeth. Tooth #47 was tender to percussion but responded normally to palpation and bite tests. Periodontal probing generally ranged from 2 to 3 mm, without any increase in tooth mobility. The health of the surrounding mucogingival tissues was within normal limits. Radiographic assessment commenced by systematic evaluation of the already available panoramic radiograph (Figure 1). The obtained preoperative periapical radiographs (Figures 2(a) and 2(b)) demonstrated the presence of one large canal along with one conical root and slight widening of the periodontal ligament space around the apex of tooth #47. A diagnosis of symptomatic irreversible pulpitis with symptomatic apical periodontitis of tooth #47 was established, and root canal treatment followed by composite buildup and crown were planned. Prior to treatment, CBCT (Planmeca Promax, Planmeca, Finland) was obtained in order to enable three-dimensional assessment of pulp space morphology. Further evaluation of sagittal, coronal, and axial slices confirmed the presence of one large round-oval canal extending from the orifice level to the root apex without any evidence of additional canals (Figures 2(a)–2(g)).

**Figure 3:**
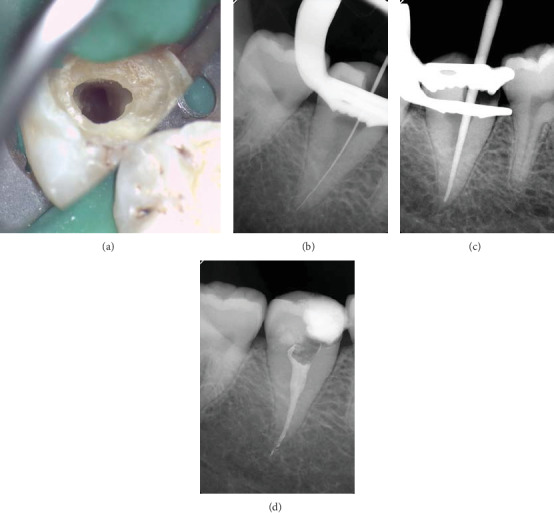
(a) Accessed cavity preparation shows the presence of one large root canal located in the center of the pulp chamber. (b) Working length determination radiograph. (c) Master gutta-percha cone radiograph. (d) Final radiograph showing dense obturation with sealer puffs.

Local anesthesia was administered through inferior alveolar nerve block using one carpule (1.8 ml) of 2% lidocaine with 1 : 100,000 of epinephrine (Lidocaine HCl, Huons Co., Seoul, Korea). Another carpule was added for buccal and lingual infiltrations. Following rubber dam isolation, caries excavation and access cavity preparation were accomplished using size 6 long shank round bur in a high-speed handpiece. Then, pulp extirpation was performed using barbed broach. The dental operating microscope (OPMI Pico, Carl Zeiss Surgical, Oberkochen, Germany) showed the presence of a large round canal orifice located in the center of the pulp chamber floor (Figure 3(a)). Following irrigation with 5.25% NaOCl, shaping of the coronal two-thirds of the canal was performed using ProTaper Gold rotary files (Dentsply Maillefer) in the following sequence: SX, S1, and S2. Working length (WL) was determined using a size 20 K file attached to an electronic apex locator and then confirmed using a digital radiograph (Figure 3(b)). After that, size 10 and 15 K files were precurved and placed against the canal walls to further explore the internal anatomy. Root canal shaping was then completed using hybrid instrumentation technique. Canal shaping to WL was first accomplished using S1 and S2 instruments. After that, apical enlargement was performed using Vortex Blue rotary files (Dentsply Tulsa Dental Specialties, Tulsa, OK) in the following sequence: 25/0.06, 30/0.06, 35/0.06, and 40/0.06. During the shaping procedure, EDTA gel (Glyde File Prep; Dentsply Maillefer, Ballaigues, Switzerland) was utilized as lubricant, and 5.25% NaOCl solution was used to irrigate the canal. Recapitulation and verification of canal patency were frequently performed during the procedure. Final irrigation was performed using 17% EDTA (Meta Biomed Co. Ltd., Mandaluyong, Korea), after which the canal was dried using sterile absorbent paper points. The snug fit of a size 40, taper 0.06 master gutta-percha cone was evaluated radiographically (Figure 3(c)). The canal was then obturated with gutta-percha cone and AH26 sealer (Dentsply Maillefer) using a hybrid technique combing cold lateral and warm vertical compaction techniques (Figure 3(d)). The pulp chamber was then cleaned using alcohol-moistened cotton pellet, after which the access cavity was temporized with Cavit. After one week, the patient reported complete resolution of the symptoms. The tooth was restored using post-retained composite buildup and then referred for the fabrication of a full coverage restoration. The patient gave his informed consent for the publication of this case.

## 3. Discussion

This case report describes the endodontic management of a mandibular second molar tooth with a single root canal aided by the contemporary advancements in the field. Assessment of preoperative periapical radiographs demonstrated the presence of one root with a large canal space suggesting the likelihood of C-shaped canal configuration. In fact, when only one root is present, the root canal system may contain only a single large canal or two root canals that may or may not join within the canal system or a C-shaped canal configuration [18]. However, systematic evaluation of CBCT slices revealed the presence of one large round canal. This observation was confirmed upon access cavity preparation as careful inspection of the pulpal floor with the dental operating microscope demonstrated the presence of one round orifice.

Cone beam computed tomography (CBCT) is a reliable and noninvasive approach that is often used in the diagnosis and treatment plan of endodontic cases. The American Association of Endodontists and American Academy of Oral and Maxillofacial Radiology published a joint position statement related to the use of CBCT [19]. The need for CBCT should be considered if the evaluation of differently angled periapical radiographs fails to provide conclusive information or if additional information in the buccolingual dimension is required. In cases deemed appropriate for the acquisition of CBCT scan, a narrow field of view which is associated with reduced radiation dose and higher spatial resolution is recommended [19]. The statement states that CBCT should only be used as an adjunctive tool in certain situations such as assessment of teeth with complex or unusual root canal anatomy, identification of calcified canals, evaluation of the outcome of endodontic treatment, and planning of surgical retreatment. Other indications for CBCT acquisition include the diagnosis and management of dentoalveolar trauma, resorptive defects, and dental implants [14, 19, 20].

Previous studies on the root canal anatomy of mandibular second molars confirm the rarity of our reported case. For instance, such observation was not documented in previous studies among American [8], Turkish [21], Thai [22], Malaysian [23], and even Saudi Arabian populations [24]. Furthermore, Weine et al., Demirbuga et al., and Gulabivala et al. reported a low prevalence rate of 1.3%, 2.1%, and 2.2% among American, Turkish, and Burmese populations, respectively [9, 13, 25]. Rahimi et al. evaluated root canal configuration of mandibular second molars among Iranian subpopulations using a clearing technique. In their study, only 6 out of the 139 teeth (4.3%) demonstrated the presence of the Vertucci Type I configuration discussed in this case report [26]. Interestingly, Fava et al. published a case report documenting the presence of one root and one root canal in all maxillary and mandibular second molar teeth of the same patient [11]. The information obtained from the different studies on root canal anatomy of teeth has important clinical implications. Whilst some of the endodontic procedural errors are encountered during the search for missing or additional root canals, such mistakes can be minimized if the clinician has an awareness of the expected location and dimensions of the pulp chamber as well as an understanding of the usual and less frequent root canal configurations. Although additional canals are more common, the clinician should also be aware that in certain situations; there is a probability of fewer root canals than the normally presumed pulp space morphology. This shall minimize the risk caused by injudicious removal of tooth structure and its subsequent effect on the mechanical properties of the tooth.

Recently, several studies have investigated the root canal anatomy of mandibular second molar teeth using CBCT. For instance, Pan et al. examined the root canal morphology in a Malaysian subpopulation and reported that the most common configuration seen in two-rooted mandibular second molars was one distinct distal canal (96.9%) and two separate mesial canals (78.1%) [23]. Furthermore, Mashyakhy and Gambarini evaluated root canal morphology differences between genders in a Saudi Arabian population. In agreement with previously cited studies, the most common configuration seen in mandibular second molars was three root canals in a two-rooted tooth whilst the prevalence of C-shaped canal configuration was found to be 8% of examined teeth. In addition, no significant differences were detected between both genders in terms of number of roots, number of canals, Vertucci types in mesial/distal roots, and prevalence of C-shaped canal configuration [24].

Sabala et al. reported that the rarer the aberration, the more likely that it was bilateral [27]. Moreover, Mashyakhy et al. assessed the bilateral symmetry of roots and root canal systems of mandibular first molars in Saudi Arabian population using CBCT. Their symmetrical analysis revealed 100% symmetry in the number of roots and 56% in the number of root canals between the right and left teeth in the same person [28]. Hence, clinicians should suspect the presence of a similar anatomic configuration on the contralateral tooth when examining the preoperative radiograph of a particular case. In our case, however, careful inspection of a magnified panoramic radiograph has clearly shown the presence of a two-rooted mandibular second molar on the contralateral side.

## 4. Conclusion

This case report presented the endodontic management of unusual root canal configuration of a mandibular second molar, a single root canal from an orifice to an apex, aided by the contemporary digital advancements in endodontics. This report highlighted the importance of textbook knowledge, radiographic examination, and careful intraoperative exploration as the main cornerstones in investigating pulp space anatomy. Clinicians need to employ the diagnostic and therapeutic tools available at their disposal in order to optimize the quality of care provided to their patients. Furthermore, clinicians should be aware of the various root canal configurations of each tooth, as it may impact subsequent treatment procedures as well as the long-term outcome of the case.
